# Diagnostic yield of chromosomal microarray and trio whole exome sequencing in congenital brain anomalies

**DOI:** 10.1192/j.eurpsy.2023.1879

**Published:** 2023-07-19

**Authors:** E. A. Fonova, A. A. Kashevarova, M. E. Lopatkina, A. A. Sivtsev, A. A. Zarubin, V. V. Demeneva, G. N. Seitova, L. I. Minaycheva, O. A. Salyukova, S. V. Fadyushina, V. V. Petrova, E. O. Belyaeva, L. P. Nazarenko, I. N. Lebedev

**Affiliations:** Research Institute of Medical Genetics, Tomsk National Research Medical Center, Tomsk, Russian Federation

## Abstract

**Introduction:**

The deductive method: from karyotyping to aCGH and WES is an important aspect in the diagnosis and search for the causes of intellectual disability due to congenital brain anomalies. There is recommendation to exclude the presence of CNV or monogenic variants for patients with a normal karyotype, but with a clinical picture of syndromic disease.

**Objectives:**

Improvement of diagnosis of intellectual disability.

**Methods:**

aCGH with 60K Agilent microarrays, WES with SureSelect Human All Exon V8

**Results:**

Pathogenic or potentially pathogenic CNVs were excluded previously by aCGH for 10 families (total 32 people, 2 families had 2 children) with intellectual disability and congenital brain anomalies (for example, polymicrogyria, pachygyria, lissencephaly). The WES identified candidate variants for all families that can lead to impaired neurodevelopment, including 3 pathogenic variants in 3 families, 3 likely pathogenic in three other families, and 10 variants with uncertain clinical significance for 4 families. Almost all of these variants were identified *de novo*, except for one family, where the proband has been a compound heterozygous for two variants in the *RELN* gene. The first case of pathogenic mutation *de novo* was detected in a girl with agenesis of the corpus callosum. It was a missense mutation DYNC1H1 (NM_001376.5): c.4868G>A (p.Arg1623Gln), which leads to impaired intellectual development in autosomal dominant type 13 (OMIM 614563). The second variant was detected in a boy with corpus callosum agenesis, pontine hypogenesis, pachygyria in the frontal lobes. It was a missense variant MACF1 (ENST00000567887.5): c.21989A>G(p.Asp7330Gly), which leads to lissencephaly 9 with complex brainstem malformation (OMIM 614563). The third variant was found in a girl with epilepsy and impaired myelination of the white matter of the parietal-occipital areas of the cerebral hemispheres. It was a missense variant CDKL5 (NM_001323289.2):c.404-1G>A that leads to developmental and epileptic encephalopathy 2 (OMIM 300672).

**Conclusions:**

Sixteen candidate variants potentially responsible for mental health were reported in this study. Most of these variants were missense changes in genes. All except one anomalies arisen *de novo.* Trio-based WES has been shown to be an important step in making a genetic diagnosis if other chromosomal and subchromosomal abnormalities had been excluded. The clinical description of the patient is the most important step for the correct interpretation of WES results, which allows to establish the exact genetic cause of the disease if several variants with unclear clinical significance were previously identified.

This study was supported by the Russian Science Foundation, grant 21-65-00017, https://rscf.ru/project/21-65-00017/

**Disclosure of Interest:**

None Declared

